# Treatment effects after maxillary expansion using invisalign first system vs. acrylic splint expander in mixed dentition: a prospective cohort study

**DOI:** 10.1186/s12903-023-03312-4

**Published:** 2023-08-27

**Authors:** Lanxin Lu, Lingling Zhang, Chengri Li, Fang Yi, Lei Lei, Yanqin Lu

**Affiliations:** 1 Xiangya Stomatological Hospital & Xiangya School of Stomatology, Central South University, 410008 Changsha, Hunan China; 2 Hunan Key Laboratory of Oral Health Research, Changsha, China; 3 Hunan Clinical Research Center of Oral Major Diseases and Oral Health, Changsha, China; 4 The Department of Dermatology, Xiangya Hospital, Central South University, Changsha, China; 5 National Engineering Research Center of Personalized Diagnostic and Therapeutic Technology, Changsha, China; 6 Department of Orthodontics, Hunan Key Laboratory of Oral Health Research & Hunan Clinical Research Center of Oral Major Diseases and Oral Health & Xiangya Stomatological Hospital, Xiangya School of Stomatology, Central South University, 410008 Changsha, Hunan China

**Keywords:** Invisalign First System, RME, Maxillary arch expansion, Natural growth, Propensity score matching, Mixed dentition

## Abstract

**Background:**

Invisalign First System (First) is a new type of orthodontic appliance for maxillary arch expansion in mixed dentition children. Till now, few studies have evaluated the expansion effects of First versus other appliances. What’s more, most studies of arch expansion did not include a natural group to rule out growth effects. This prospective cohort study aimed to evaluate the dental and dentoalveolar effects using First or acrylic splint rapid maxillary expander (RME) in adolescents excluding growth factors.

**Materials and methods:**

After screening by strict inclusion criteria and propensity score matching (PSM), fifty-one patients were included: First group (n = 17), RME group (n = 17), and natural growth (NG) group (n = 17). Nine indicators including dental arch width, dentoalveolar arch width, and inclination of the molars were measured on digital dental casts at baseline (T0) and six-month follow-up (T1). Paired *t*-tests were used for intra-group results, and two-sample independent *t*-tests were used for inter-group comparisons.

**Results:**

There was no significant increase in all indicators within six months in the NG group (*p* > 0.05). In the First group and RME group, all width indicators were significantly increased after treatment (*p* < 0.05). The RME group exhibited greater expansion than the First group in intercanine width, first interpremolar width, second interdeciduous molar width, first intermolar width, arch perimeter, intercanine dentoalveolar width, intermolar dentoalveolar width, and inclination of the molars (*p* < 0.05). Whereas, there was no significant difference in arch depth between the two treated groups.

**Conclusions:**

Both First and RME can expand the maxillary arch in mixed dentition. In case of mild to moderate maxillary transverse deficiency (MTD), Invisalign First System could be a reasonable option. RME shows significant better efficiency of dental arch expansion than First, recommended for patients with severe MTD.

**Trial registration:**

This prospective study was registered on ClinicalTrials.gov (01/02/2022, registration number: ChiCTR2200056220). The trial was approved by the Ethical Committee of the Hunan Xiangya Stomatological Hospital Central South University (20,200,088), and informed consent was obtained from all subjects and their legal guardian(s).

**Supplementary Information:**

The online version contains supplementary material available at 10.1186/s12903-023-03312-4.

## Introduction

Maxillary transverse deficiency (MTD) is one of the most pervasive skeletal problems in orthodontics, with a prevalence of 21% in patients during mixed dentition [1, 2]. Traditionally, acrylic splint rapid maxillary expander (RME) has been used as a proven method for treating MTD [3]. The effects of RME on the maxillary arch have been extensively investigated. According to the study of Geran et al., an acrylic splint expander is an effective approach to correct the transverse deficiency in mixed dentition [4]. But the cumulative force that RME normally passes through the midpalatal suture is approximately 100 N [5]. Thus, some researchers are concerned about the unwanted consequences of these heavy forces on the periodontal alveolar bone, for example by causing bone dehiscence and gingival recession [6, 7]. At the same time, discomfort and difficulties in cleaning teeth often arise when patients use the RME [8].

The Invisalign First System (First) is a new option designed specifically for patients in mixed dentition. It can be used in patients between 6 and 10 years old with a crossbite, crowding, and a narrow maxillary arch in particular. According to a previous study, it is more comfortable, aesthetic, and more convenient to maintain oral health [9]. And many clinicians have tried to use it to treat MTD in mixed dentition. However, its effectiveness has not been thoroughly explored [10, 11]. Blevins et al. were the first to report the effectiveness of First in maxillary arch expansion[12]. Its validity in widening arch width in patients with mixed dentition was also demonstrated by Levrini et al. [2]. But these studies have some limitations to obtain more systematic and reliable results: (i) lack of control group; (ii) small sample size.

Till now, there has been no prospective cohort study analyzing the dental and dentoalveolar effects after maxillary expansion using Invisalign First System versus acrylic splint expander in growing subjects. Thus, clinicians have to rely on their experience and low-quality evidence when developing treatment plans. What’s more, previous research on arch expansion during mixed dentition lacked observation of blank controls to exclude the influence of growth. Hence, systematic and scientific clinical research is needed to evaluate the effectiveness of First in maxillary arch expansion.

Many confounding factors in clinical research can affect the experiment’s reliability. Propensity score matching (PSM) is a methodology for reducing bias due to observed covariates in studies for causal effect [13]. PSM has been proven an effective method to balance baseline data between groups [14]. It has been widely used in many clinical studies to compare the treatment effects and clinical prognosis of different treatment options [15, 16]. But the use of PSM in orthodontic clinical studies is still uncommon.

Therefore, the purpose of this prospective cohort study was to evaluate the dental and dentoalveolar effects using First or RME in mixed dentition excluding growth factors.

## Materials and methods

### Participants

The inclusion criteria were as follows:

1. mixed dentition with first molars fully erupted;
2. posterior transverse discrepancy between maxillary and mandibular arches ≤ 5 mm;
3. mild or moderate crowding;
4. prepubertal stage of development (CS1–CS3 in cervical vertebral maturation) [17].

Exclusion criteria included:

1. Class III malocclusion;
2. previous orthodontic treatment;
3. congenitally missing teeth;
4. disturbance syndrome of the temporomandibular joint;
5. cleft lip and palate;
6. use of additional orthodontic devices during the observation period.

### Propensity score matching (PSM)

The initial treated sample consisted of 70 patients following the inclusion and exclusion criteria, including 40 patients treated with RME, and 30 patients treated with Invisalign First System. Propensity score matching (PSM) was used to screen the final sample from the initial sample. The ABO discrepancy index (DI), cervical vertebral maturation (CVM), sex, and Angle’s classification were analyzed and recorded as confounding factors for each patient in the two treated groups.

DI was used to quantify the severity of malocclusion, which takes into account overjet, overbite, anterior open bite, lateral open bite, crowding, occlusal relationships, lingual posterior crossbite, buccal posterior crossbite, cephalometric items, and other items [18]. The initial sample was divided into three levels of severity based on the pre-treatment DI: low (DI < 7), medium (DI 8–16), and high complexity (DI ≥ 17) [19]. CVM is also quantified according to the pre-treatment cephalometric film, which is divided into six stages [17]. The gender of the patient, as well as the class I or II malocclusions, were recorded as binary variables.

The multivariate logistic regression model was calculated using SPSS software. For propensity score analysis, appliance type was used as the dependent variable, while sex (male, female), DI severity (low, medium, high), CVM (CS1, CS2, CS3), and Angle’s classification (Class I, Class II) were modeled as covariates. A one-to-one nearest neighbor matching algorithm was applied using SPSS software, and a caliper with a width of 0.02 times the standard deviation of the logit of the propensity score was applied as the matching criteria [20]. The final treated sample of 34 patients was obtained after PSM, including 17 patients in the First group, and 17 patients in the RME group. A χ^2^ test was used to compare the baseline data between the two groups, controlling the balance of covariates (Table S1).

Seventeen children aged from 6 to 10 years old who were routinely observed by the researchers for natural growth and did not have any orthodontic treatment were included in the NG group.

### Treatment protocol

A total of 51 patients were included in this study: First group (n = 17), RME group (n = 17), and NG group (n = 17). They were treated or observed in the Hunan Xiangya Stomatological Hospital Central South University, from February 2022 to February 2023.

The acrylic splint expander was utilized in the RME group (Fig. S1). The patients underwent a standardized protocol of RME with two turns a day (~ 0.25 mm per turn) until the expansion screw reached 7 mm (about 14 days). The RME was kept on the teeth as a passive retainer and oral scans of subjects were obtained 6 months after treatment. Patients were instructed to have an appointment every month and re-bond if the RME became loose, therefore, the oral scan time is delayed accordingly.

Patients in the First group were treated with Invisalign First System (Fig. S2). The ClinCheck for each patient was planned with the same standardized expansion protocol: the maxillary arch is expanded by first moving the molars and then simultaneously expanding all the posterior deciduous teeth and canines. All patients were required to wear their aligners all day except for meals and tooth brushing. Patients changed aligners every 7 days and every 2 months the clinician checked the good aligner fit and the position of the attachments to check that they were all in place. Patient compliance was noted in the clinical records and was appraised with a 3-point Likert-type scale (poor, moderate, good) [21]. Only patients with “good” ratings were included in this study. In case of tooth loss and eruption, the patient will use a new scan to re-produce the aligner and continue with the original prescription until the same final position as in the first approved ClinCheck plan. The average time from the initial scan to the final scan was 6 months.

### Sample size calculation

Based on previous studies, the study outcome variable was set to be the change in maxillary first intermolar width [22]. The null hypothesis tested was that there was no significant difference in maxillary expansion between the RME group and the First group. Group sample sizes of 13 and 13 would achieve 81.727% power to reject the null hypothesis of equal means. The population means the difference of 1.9 was used in the calculation, with a standard deviation of 0.8 for the RME group and 2.0 for the First group. Meanwhile, the significance level (alpha) of 0.050 and a two-sample unequal-variance *t*-test were used.

### Treatment evaluation

Digital models were established by Geomagic Design 2016 software (Geomagic Company, USA) based on iTero (Align Technology Inc, USA) scans at baseline (T0) and six-month follow-up (T1). The pretreatment and posttreatment models were registered and the final superimposition of both two models was achieved to unify the measurement reference system (Fig. S3).

**Fig. 1:**
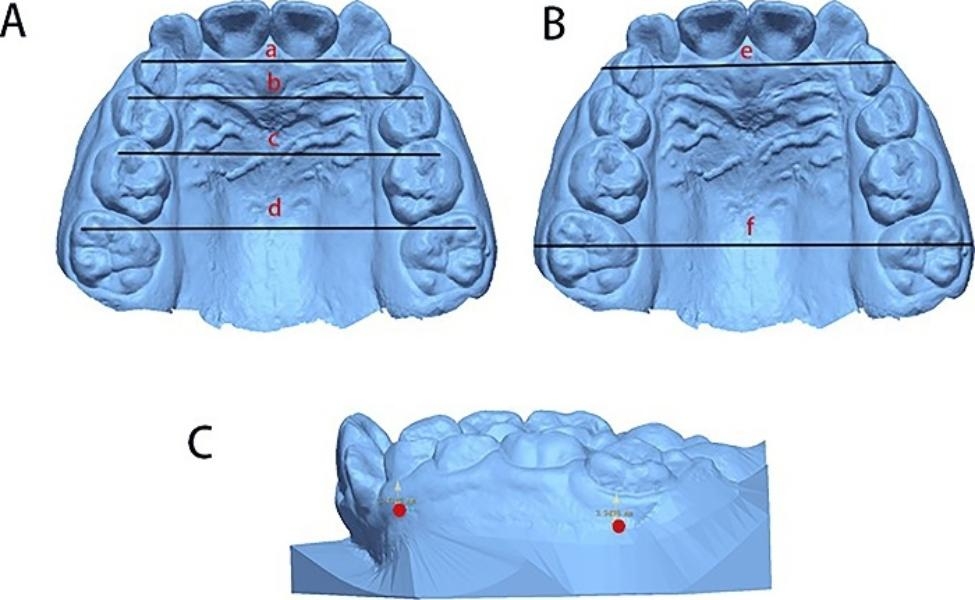
Arch width linear measurements. A. Dental indicators: (a) 53–63 width; (b) 14–24 width; (c) 55–65 width; (d) 16–26 width. B. Dentoalveolar indicators: (e) intercanine dentoalveolar width; (f) intermolar dentoalveolar width. C. Dentoalveolar marker points

**Fig. 2:**
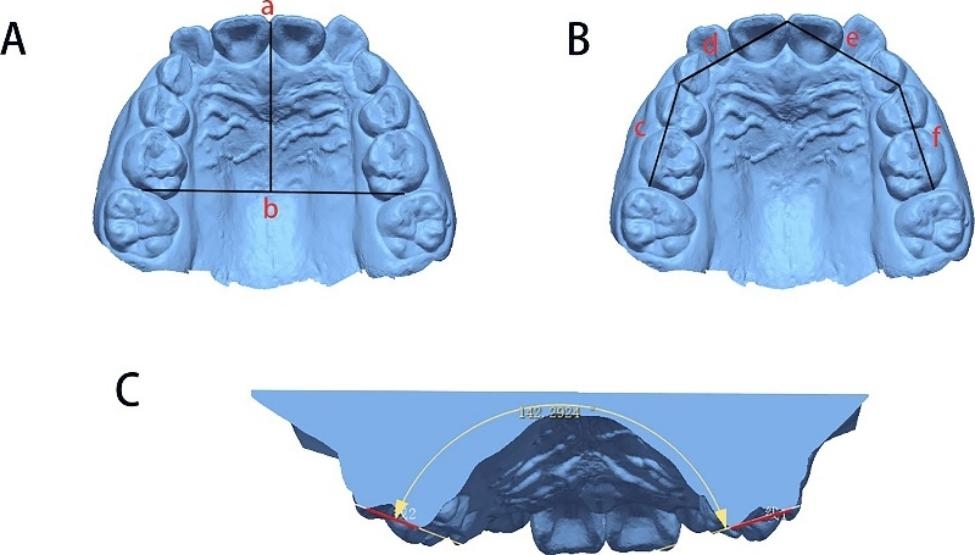
**A**: Arch depth (from a to b). **B**: Arch perimeter (from c to f). **C**: Molar inclination

The following indicators were measured on the maxillary arch as described in Figs. 1 and 2:

1. Intercanine width (53–63): linear distance between cusp tips of the deciduous canines [23];
2. First interpremolar width (14–24): linear distance between the buccal cusp tips of the first premolars [23];
3. Second interdeciduous molar width (55–65): linear distance between the mesiobuccal cusp tips of the second deciduous molars [23];
4. First intermolar width (16–26): linear distance between the mesiobuccal cusp tips of the first molars [23];
5. Arch depth: length of perpendicular line constructed from contact point between mesial contact points of central incisors to line connecting contact points between second deciduous molars and first permanent molars [4];
6. Arch perimeter: line from the mesial contact point of 1 molar through mesial and distal contact points of 6 anterior teeth to mesial contact point of opposite molar [4];
7. Intercanine/Intermolar dentoalveolar width: from the most prominent buccal bulge on the alveolus superior to the maxillary first molar/canine. This measure was usually 3 to 5 mm superior to the gingival crest [6];
8. Inclination of the molars: The angle formed by the intersecting lines drawn across the mesial buccal and mesial lingual cusp tips of both the right and left first molars was defined by Handelman et al. as the maxillary first molar inclination [6].

### Statistical analysis

Data analysis was performed using IBM SPSS Statistics version 26.0 (IBM Corp., Armonk, NY, USA), and statistical significance was set at *p* < 0.05. All measurements were repeated twice over 2 weeks by the same observer. The interclass correlation coefficient was calculated to assess systemic intra-examiner errors between the two measurements, and there were no significant differences between the two measurements. The Shapiro-Wilk test was used to examine the normality between the measurements at T0 and T1. Paired *t*-tests were used to compare the indicators before and after treatment within groups. Two-sample independent *t*-tests were used for comparisons between groups.

## Results

### Final sample

The PSM and baseline data for the final sample are shown in Table S1. The χ^2^ tests showed that the distributions of sex, CVM stage, DI score, and Angle’s classification were not significantly different between the two treated groups (*p* > 0.05). In the final sample, the sample size for both groups was 17.

### Intra-group comparisons

#### Comparisons within the first group

The changes of all the detected markers from T0 to T1 in the First group were displayed in Table 1. There was an increase in all dental and dentoalveolar width indexes after treatment, and the changes were statistically significant. For the dental indicators, the biggest increase was observed in 14–24 width (2.83 ± 1.90 mm; *p* < 0.05), followed by 16–26 width (2.43 ± 1.42 mm; *p* < 0.05), 55–65 width (1.93 ± 1.75 mm; *p* < 0.05), and 53–63 width (1.89 ± 1.56 mm; *p* < 0.05). In regards to dentoalveolar measures, it was observed that intermolar dentoalveolar width (1.43 ± 0.86 mm; *p* < 0.05) exhibited a greater increase than intercanine dentoalveolar width (1.05 ± 1.14 mm; *p* < 0.05). Additionally, the arch perimeter increased after treatment, and the changes were statistically significant (1.69 ± 2.01 mm; *p* < 0.05). Conversely, there was a statistically significant decrease in arch depth (-0.46 ± 0.86 mm; *p* < 0.05) and buccal inclination of the molars (-4.49 ± 6.87°; *p* < 0.05).

### Comparisons within the RME group

In the RME group, there was a significant increase in all dental and dentoalveolar width indexes after treatment. (Table 1) The greatest increase in maxillary dental width was found in 55–65 width (5.52 ± 1.31 mm; *p* < 0.05), followed by the 16–26 width (5.32 ± 1.13 mm; *p* < 0.05), 14–24 width (5.05 ± 1.81 mm; *p* < 0.05), and 53–63 width (4.18 ± 2.01 mm; *p* < 0.05). Additionally, the intercanine dentoalveolar width (2.65 ± 2.61 mm; *p* < 0.05), intermolar dentoalveolar width (4.01 ± 1.08 mm; *p* < 0.05), and arch perimeter (3.32 ± 1.86 mm; *p* < 0.05) exhibited a significant increase. Instead, the measurements conclude a significant buccal inclination of the molars (-9.64 ± 7.02°; *p* < 0.05). However, no statistically significant change in arch depth was observed.

### Comparisons within the NG group

As exhibited in Table 1, a slight increase was observed in all detected indicators in the NG group, but the increase was not statistically significant (*p* > 0.05).

**Table 1:** Paired *t-*test comparisons of arch dimensional changes in the First group, RME group, and NG group

	First group				RME group				NG group		
	T0	T1			T0	T1			T0	T1	
	M ± SD	M ± SD	*P*		M ± SD	M ± SD	*p*		M ± SD	M ± SD	*p*
53–63, mm	32.74 ± 2.11	34.64 ± 2.80	0.001*		33.56 ± 2.64	37.74 ± 1.95	0.001*		33.28 ± 2.59	33.40 ± 2.74	0.563
14–24, mm	38.31 ± 1.98	41.14 ± 2.56	0.001*		38.85 ± 3.54	43.90 ± 3.74	0.001*		39.60 ± 1.82	39.83 ± 1.59	0.081
55–65, mm	43.64 ± 1.61	45.56 ± 2.55	0.001*		43.72 ± 3.46	49.24 ± 2.70	0.001*		45.18 ± 1.72	45.34 ± 1.64	0.060
16–26, mm	50.26 ± 2.42	52.70 ± 2.47	0.001*		49.95 ± 2.81	55.28 ± 3.33	0.001*		50.88 ± 2.34	51.14 ± 2.25	0.092
Arch depth, mm	27.61 ± 2.56	28.07 ± 2.91	0.041*		28.92 ± 2.54	28.56 ± 2.38	0.065		28.11 ± 1.95	28.36 ± 1.87	0.063
Arch perimeter, mm	74.28 ± 5.21	75.97 ± 4.84	0.003*		76.61 ± 3.86	79.93 ± 3.26	0.001*		75.44 ± 3.10	75.77 ± 2.99	0.067
Intercanine dentoalveolar width, mm	36.39 ± 2.36	37.44 ± 2.16	0.002*		36.61 ± 2.31	39.26 ± 2.10	0.001*		36.68 ± 2.38	37.18 ± 2.06	0.056
Intermolar dentoalveolar width, mm	58.37 ± 2.30	59.80 ± 2.49	0.001*		57.84 ± 2.79	61.84 ± 2.57	0.001*		58.92 ± 3.04	59.02 ± 3.15	0.469
Inclination of the molars, °	156.68 ± 12.01	152.19 ± 8.90	0.016*		154.40 ± 8.48	144.76 ± 10.07	0.001*		148.34 ± 11.32	149.70 ± 11.42	0.138

T0, pre-treatment; T1, 6 months after treatment; First, Invisalign First System; RME, acrylic splint rapid maxillary expander; NG, natural growth; 53–63, intercanine width; 14–24, first interpremolar width; 55–65, second interdeciduous molar width; 16–26, first intermolar width; *p < 0.05 indicates statistical significance

### Inter-group comparisons

#### First group vs. RME group

When compared with the RME group, less expansion was obtained in the First group in all dental and dentoalveolar width indexes after treatment, and the differences were statistically significant (Table 2). Less increase was found in the First group in 16–26 width (-2.89 ± 0.44 mm; *p* < 0.05), 53–63 width (-2.28 ± 0.68 mm; *p* < 0.05), 14–24 width (-2.22 ± 0.68 mm; *p* < 0.05), and 55–65 width (-3.59 ± 0.54 mm; *p* < 0.05). In terms of dentoalveolar expansion, less increase at the canine level (-1.60 ± 0.69 mm; *p* < 0.05) and the molar level (-2.57 ± 0.33 mm; *p* < 0.05) was demonstrated. Also, a less increase in arch perimeter (-1.63 ± 0.66 mm; *p* < 0.05) was observed after treatment in the First group. But there was no statistically significant difference between the two groups in the arch depth changes. Interestingly, less molar buccal tipping was shown in the First group than the RME group (5.14 ± 2.38°; *p* < 0.05).

**Table 2:** *t*-Test comparisons of arch dimensional changes between the First group vs. the RME group

Variables	First group			RME group				Difference	*p* Value	95% CI of the Difference	
	Mean	SD		Mean		SD				Lower	Upper
53–63, mm	1.89	1.56	4.18		2.01		-2.28		0.002*	-3.67	-0.88
14–24, mm	2.83	1.90	5.05		1.81		-2.22		0.003*	-3.61	-0.83
55–65, mm	1.93	1.75	5.52		1.31		-3.59		0.000*	-4.70	-2.49
16–26, mm	2.43	1.42	5.32		1.13		-2.89		0.000*	-3.78	-1.99
Arch depth, mm	-0.46	0.86	-0.36		0.75		-0.10		0.715	-0.67	0.46
Arch perimeter, mm	1.69	2.01	3.32		1.86		-1.63		0.020*	-2.98	-0.28
Intercanine dentoalveolar width, mm	1.05	1.14	2.65		2.61		-1.60		0.028*	-3.02	-0.19
Intermolar dentoalveolar width, mm	1.43	0.86	4.01		1.08		-2.57		0.000*	-3.25	-1.89
Inclination of the molars, °	-4.49	6.87	-9.64		7.02		5.14		0.038*	0.29	10.00

First, Invisalign First System; RME, acrylic splint rapid maxillary expander; NG, natural growth; 53–63, intercanine width; 14–24, first interpremolar width; 55–65, second interdeciduous molar width; 16–26, first intermolar width; *p < 0.05 indicates statistical significance

### First group vs. NG group

A greater increase in all dental and dentoalveolar width indexes was shown in the First group compared to the NG group, and the differences were statistically significant (Table 3). Greater increase was found in the First group in 53–63 width (1.77 ± 0.45 mm; *p* < 0.05), 14–24 width (2.54 ± 0.50 mm; *p* < 0.05), 55–65 width (1.77 ± 0.43 mm; *p* < 0.05), 16–26 width (2.17 ± 0.37 mm; *p* < 0.05), intermolar dentoalveolar width (1.33 ± 0.25 mm; *p* < 0.05) and intercanine dentoalveolar width (0.73 ± 0.33 mm; *p* < 0.05). A greater increase in arch perimeter (1.37 ± 0.51 mm; *p* < 0.05) was also detected in the First group, and a greater reduction in arch depth (-0.70 ± 0.24 mm; *p* < 0.05) was observed. In addition, more buccal inclination of the molars was found in the First group (-5.84 ± 1.88°; *p* < 0.05).

**Table 3:** *t*-Test comparisons of arch dimensional changes between the First group vs. the NG group

Variables	First group			NG group				Difference	*p* Value	95% CI of the Difference	
	Mean	SD		Mean		SD				Lower	Upper
53–63, mm	1.89	1.60	0.12		0.87		1.77		0.000*	0.85	2.69
14–24, mm	2.83	1.90	0.29		0.54		2.54		0.001*	1.53	3.56
55–65, mm	1.93	1.75	0.16		0.32		1.77		0.000*	0.89	2.66
16–26, mm	2.43	1.42	0.27		0.61		2.17		0.000*	1.41	2.94
Arch depth, mm	-0.46	0.86	0.24		0.51		-0.70		0.007*	-1.19	-0.20
Arch perimeter, mm	1.69	2.01	0.32		0.68		1.37		0.012*	0.32	2.42
Intercanine dentoalveolar width, mm	1.05	1.14	0.32		0.75		0.73		0.034*	0.06	1.41
Intermolar dentoalveolar width, mm	1.43	0.86	0.10		0.54		1.33		0.000*	0.83	1.84
Inclination of the molars, °	-4.49	6.87	1.35		3.57		-5.84		0.004*	-9.67	-2.02

First, Invisalign First System; RME, acrylic splint rapid maxillary expander; NG, natural growth; 53–63, intercanine width; 14–24, first interpremolar width; 55–65, second interdeciduous molar width; 16–26, first intermolar width; *p < 0.05 indicates statistical significance

### RME group vs. NG group

The changes in 53–63 width (4.05 ± 0.53 mm; *p* < 0.05), 14–24 width (4.76 ± 0.47 mm; *p* < 0.05), 55–65 width (5.36 ± 0.33 mm; *p* < 0.05), and 16–26 width (5.05 ± 0.31 mm; *p* < 0.05) were significantly increased in the RME group compared to the NG group (Table 4). Furthermore, greater increases were found in the RME group in intermolar dentoalveolar width (3.91 ± 0.29 mm; *p* < 0.05), intercanine dentoalveolar width (2.15 ± 0.68 mm; *p* < 0.05), and the arch perimeter (3.00 ± 0.48 mm; *p* < 0.05), with greater reduction in arch depth (0.60 ± 0.22 mm; *p* < 0.05). Besides, the RME group had a greater buccal inclination of the molars (-10.99 ± 1.90°; *p* < 0.05) than the NG group.

**Table 4:** *t*-Test comparisons of arch dimensional changes between the RME group vs. the NG group

Variables	RME group			NG group				Difference	*p* Value	95% CI of the Difference	
	Mean	SD		Mean		SD				Lower	Upper
53–63, mm	4.18	2.01	0.12		0.87		4.05		0.000*	2.96	5.14
14–24, mm	5.05	1.81	0.29		0.54		4.76		0.000*	3.80	5.73
55–65, mm	5.52	1.31	0.16		0.32		5.36		0.000*	4.70	6.01
16–26, mm	5.32	1.13	0.27		0.61		5.05		0.000*	4.42	5.69
Arch depth, mm	-0.36	0.75	0.24		0.51		0.60		0.011*	-1.05	1.15
Arch perimeter, mm	3.32	1.86	0.32		0.68		3.00		0.000*	2.02	3.98
Intercanine dentoalveolar width, mm	2.65	2.61	0.32		0.75		2.15		0.004*	0.76	3.54
Intermolar dentoalveolar width, mm	4.01	1.08	0.10		0.54		3.91		0.000*	3.31	4.50
Inclination of the molars, °	-9.64	7.02	1.35		3.57		-10.99		0.000*	-14.88	-7.10

First, Invisalign First System; RME, acrylic splint rapid maxillary expander; NG, natural growth; 53–63, intercanine width; 14–24, first interpremolar width; 55–65, second interdeciduous molar width; 16–26, first intermolar width; *p < 0.05 indicates statistical significance

## Discussion

To our knowledge, this is the first prospective study evaluating the treatment effects of Invisalign First System on maxillary expansion in mixed dentition which included a natural growth group and traditional RME group. Due to the multitude of confounding factors affecting clinical outcomes in orthodontic studies, there remains a need for more systematic and scientific research to assess the effects in maxillary arch expansion using various expanders. PSM has been widely used in recent clinical research to improve experimental credibility by balancing the baseline data between groups [24, 25]. In this study, PSM was utilized innovatively to balance confounding factors such as sex, CVM stage, DI score, and Angle’s classification, thereby greatly improving the comparability between the two treated groups.

In cases of mixed dentition, there is often a natural growth in maxillary arch, which may impact the accuracy of the experiment results. To eliminate growth-related influences, the NG group was included in this study. Within the six-month observation period, no statistically significant differences were observed in the NG group, which was consistent with the results of Bishara et al., demonstrating that the spontaneous growth of the maxillary arch during this period was minimal (less than 0.5 mm) [26]. Therefore, maxillary arch changes in the two treated groups of subjects could reasonably be attributed to the intervention of the expanders.

Measuring the changes in digital models after the maxillary expansion is challenging. Finding a stable reference area to overlap the pre- and posttreatment digital models to establish a unified measurement reference system is the key solve this problem [27]. A previous review found that the medial portion of the second and third rugae, as well as the palatal dome behind this zone, appeared to be the most stable areas during orthodontic treatment and growth [28]. In our study, the same regions were used to superimpose T0 and T1 digital casts.

The advent of the Invisalign First System has provided a new option of maxillary arch expansion for teenagers seeking more aesthetically pleasing and convenient treatment [29, 30]. In our study, all indicators on dental width increased after treatment in the First group, with the greatest expansion detected in 14–24 width. The least increase was observed in 53–63 width, consistent with the findings of Lione R et al. [23]. All dentoalveolar width-related indicators also increased after treating with First comparied to the NG group. In accordance with the study of Levrini L et al. [2], we may conclude that Invisalign First System is considered effective for growing patients requiring maxillary arch expansion. However, it is noteworthy that dental widths in our study were measured using the teeth cusps, which may be affected by buccal tipping during expansion.

RME is a traditional approach that is widely used in treating transverse maxillary discrepancy [31, 32]. Typically, maxillary expansion is performed until overcorrection was achieved. In our study, the RME group underwent an expansion of 7 mm. Significant increases were found in all width indicators after treatment in the RME group. The greatest increase was observed in 55–65 width, followed by 16–26 width, 14–24 width, and 53–63 width. But the difference between the increases was minimal, indicating a near-parallel pattern of maxillary dental arch expansion. It was also recommended by other scholars that arch expansion treatment should be carried out during mixed dentition if parallel maxillary arch expansion is desired [33, 34].

For the two treated groups, RME demonstrated greater expansion with larger increases in all width indicators compared to First, indicating that it may be a preferable option for severe MTD. RME is thought to be an orthopedic appliance to produce skeletal expansion effects by the fracture of the palatal suture [35]. Whereas, the maxillary arch expansion obtained by First may be attributed to dentoalveolar remodeling [36, 37]. The arch perimeter of both groups increased, with a greater change exhibited in RME group. As was reported by Cretella et al., Invisalign First System can achieve shape modifications of the maxillary arch for aesthetic and functional purposes during expansion, which may account for the less increase in arch perimeter [38]. An increase in arch depth was observed in both treated groups, but there was no statistically significant difference between groups. Significant buccal inclination of molars were observed after treatment in both groups, which supports the viewpoint that expansion causes dental tipping. A study by Steiner et al. showed that excessive buccal tilt may lead to bone cracking, gingival recession, and reduced alveolar crest levels [39]. In this study, we found that patients in the First group showed less buccal inclination of molars than the RME group, which may owing to the additional buccal root torque pre-designed for the first molars during expansion. Thus, we may conclude that Invisalign First System may be recommended for patients with mild to moderate MTD in mixed dentition, while RME allows a greater expansion, recommended for patients with severe MTD.

Despite all the advantages, our study still had some limitations. Firstly, clinical studies with larger samples are needed to validate the experiment results. Secondly, further evaluation is needed to analyse the long-term stability of the two appliances. Last but not least, digital models were used for measurement in this study, due to concerns regarding radiation exposure of cone-beam computed tomography with 6 months.

## Conclusions

The results of this preliminary study demonstrate that both First and RME can expand the maxillary arch in mixed dentition. In case of mild to moderate MTD, Invisalign First System could be a reasonable option. RME shows significant better efficiency of dental arch expansion than Invisalign First, recommended for patients with severe maxillary transverse deficiency.

### Electronic supplementary material

Below is the link to the electronic supplementary material.

Supplementary Material 1

## Data Availability

The data of the findings in this study are available from the corresponding author upon reasonable request.

## Abbreviations

CVM: Cervical vertebral maturation
DI: ABO discrepancy index
53–63: Intercanine width
14–24: First interpremolar width
55–65: Second interdeciduous molar width
16–26: First intermolar width
First: Invisalign First System
MTD: Maxillary transverse deficiency
PSM: Propensity score matching
RME: Acrylic splint rapid maxillary expander
