# Supplementary material for: Treatment effects after maxillary expansion using invisalign first system vs. acrylic splint expander in mixed dentition: a prospective cohort study
Source: BMC Oral Health. 2023 Aug 27;23:598. doi: 10.1186/s12903-023-03312-4 (PMC10463527; doi:10.1186/s12903-023-03312-4)
Supplement: Supplementary file 1 — Supplementary Material 1 [file 12903_2023_3312_MOESM1_ESM.docx]

**Treatment effects after maxillary expansion using Invisalign First System vs acrylic splint expander in mixed dentition: a prospective cohort study**

Lanxin Lu^1,2,3,+^, Lingling Zhang^1,2,3,4,5,+^ , Chengri Li^1,2,3^, Fang Yi^1,2,3^, Lei Lei^1,2,3^, and Yanqin Lu^1,2,3,*^

^1^ Xiangya Stomatological Hospital & Xiangya School of Stomatology, Central South University, Changsha, 410008, Hunan, China

^2^ Hunan Key Laboratory of Oral Health Research, China

^3^Hunan Clinical Research Center of Oral Major Diseases and Oral Health, China

^4^ The Department of Dermatology, Xiangya Hospital, Central South University

^5^National Engineering Research Center of Personalized Diagnostic and Therapeutic Technology

^+^ Lanxin Lu and Lingling Zhang contributed equally to this article as co-first authors.

Corresponding Author:

Y. Lu, Professor, PhD.,

Department of Orthodontics, Hunan Key Laboratory of Oral Health Research & Hunan 3D Printing Engineering Research Center of Oral Care & Hunan Clinical Research Center of Oral Major Diseases and Oral Health & Xiangya Stomatological Hospital & Xiangya School of Stomatology, Central South University, Changsha, 410008, Hunan, China.

Telephone：+86 13973102658

Email: 213031@csu.edu.cn

Journal name: *BMC Oral Health*


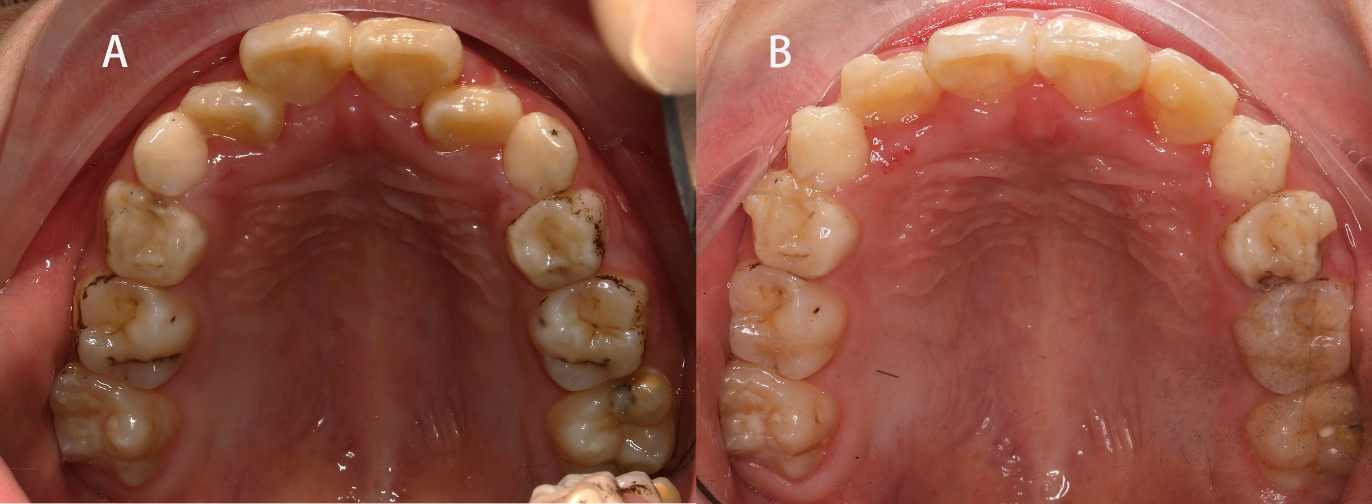


**Fig. S1** A. Maxillary arch before Invisalign First System treatment. B. Maxillary arch after 6 months of treatment with Invisalign First System.


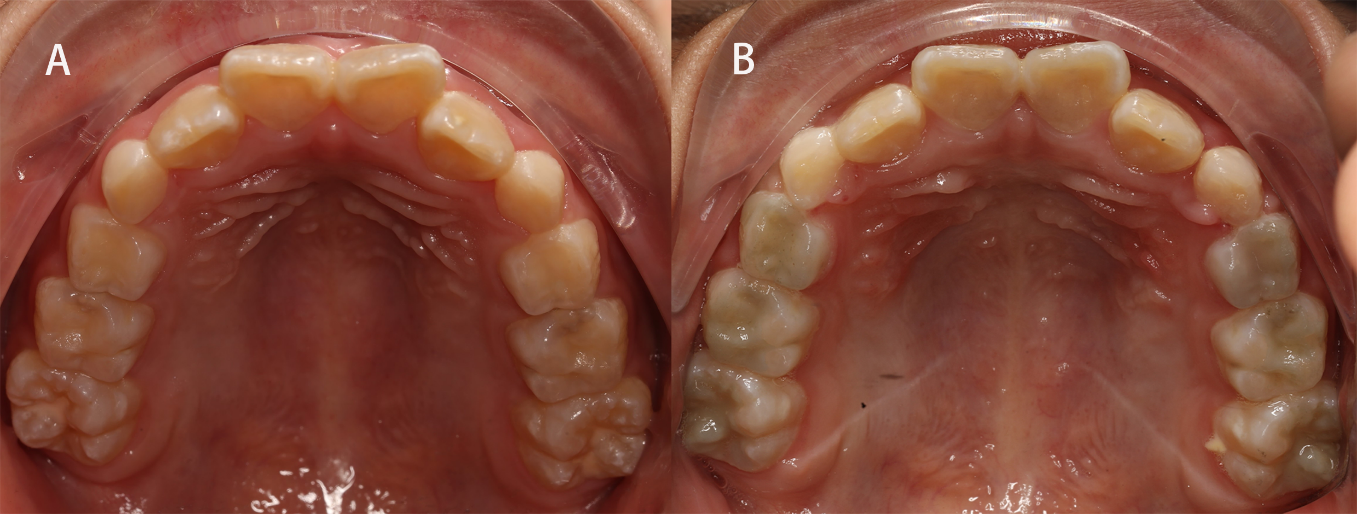


**Fig. S2** A. Maxillary arch before RME. B. Maxillary arch after 6 months of treatment with RME.


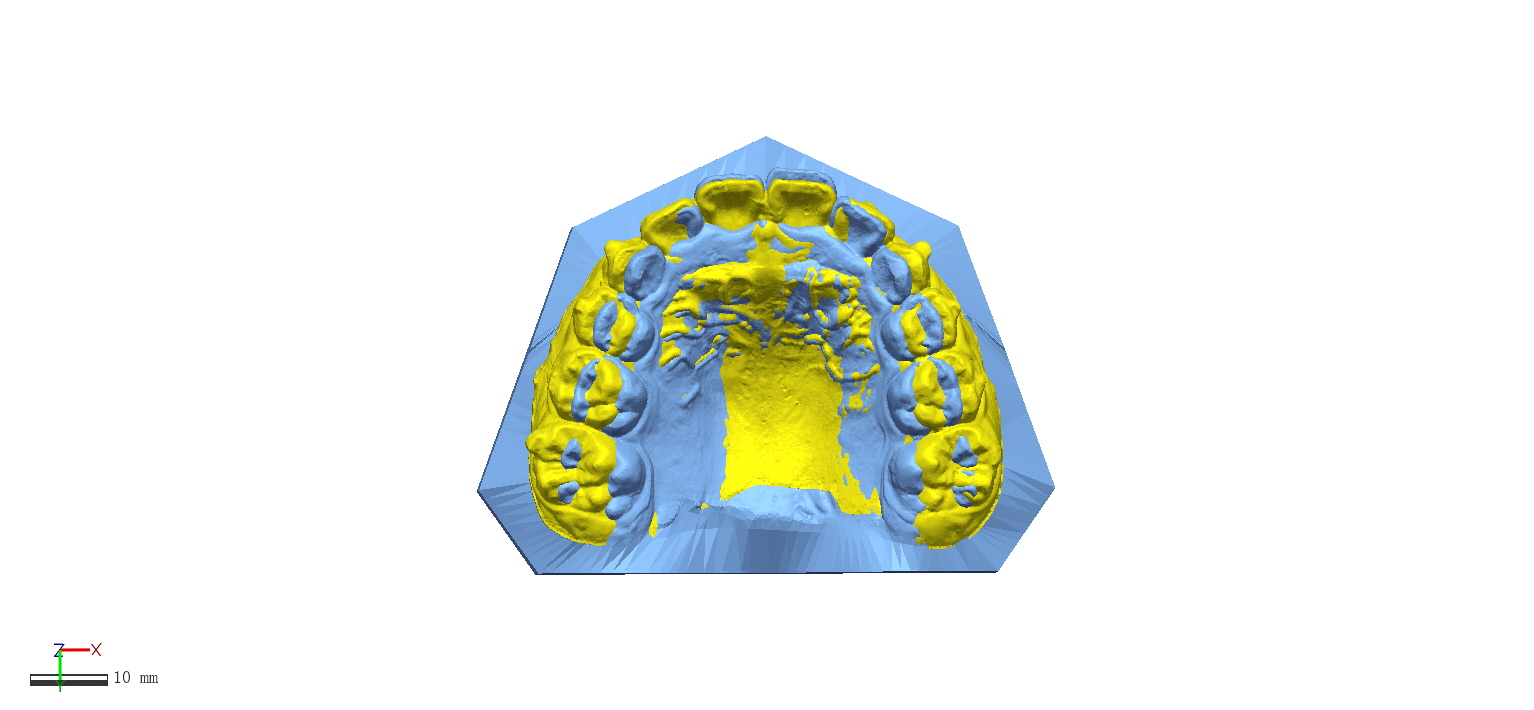


**Fig. S3** Superimposition of T0 and T1 digital models

**Table S1** Baseline data of the two treated groups of patients after PSM

|  | Baseline data after PSM | | | | | | *P* value | |
| --- | --- | --- | --- | --- | --- | --- | --- | --- |
|  | First group | | | RME group | | |  |  |
| Sex |  |  |  | |  |  | |  |
| male | 8 | | 8 | | | 1.00 | |  |
| female | 9 | | 9 | | |  |  |  |
| Age |  |  |  | |  |  | |  |
| CS1 | 7 | | 6 | | | 0.34 | |  |
| CS2 | 10 | | 8 | | |  |  |  |
| CS3 | 0 | | 3 | | |  |  |  |
| Complexity |  |  |  | |  |  | |  |
| DI low | 1 | | 1 | | | 0.84 | |  |
| DI medium | 3 | | 5 | | |  |  |  |
| DI high | 13 | | 11 | | |  |  |  |
| Angle’s classification |  |  |  | |  |  | |  |
| Class I | 5 | | 4 | | | 1.00 | |  |
| Class II | 12 | | 13 | | |  |  |  |

First, Invisalign First System; RME, acrylic splint rapid maxillary expander;

**P* < 0.05 indicates statistical significance.
